# Long-term outcomes of medical therapy versus successful recanalisation for coronary chronic total occlusions in patients with and without type 2 diabetes mellitus

**DOI:** 10.1186/s12933-020-01087-4

**Published:** 2020-07-04

**Authors:** Lei Guo, Junjie Wang, Huaiyu Ding, Shaoke Meng, Xiaoyan Zhang, Haichen Lv, Lei Zhong, Jian Wu, Jiaying Xu, Xuchen Zhou, Rongchong Huang

**Affiliations:** 1 Department of Cardiology, The First Affiliated Hospital of Dalian Medical University, Dalian, People’s Republic of China; 2 Department of Radiology, Fuyang Hospital of Anhui Medical University, Fuyang, People’s Republic of China; 3 Department of Cardiology, Capital Medical University Affiliated Beijing Friendship Hospital, Beijing, People’s Republic of China

**Keywords:** Chronic total occlusions, Diabetes, Percutaneous coronary intervention, Medical therapy, Outcomes

## Abstract

**Background:**

In this study, we compared the outcomes of medical therapy (MT) with successful percutaneous coronary intervention (PCI) in chronic total occlusions (CTO) patients with and without type 2 diabetes mellitus.

**Methods:**

A total of 2015 patients with CTOs were stratified. Diabetic patients (n = 755, 37.5%) and non-diabetic patients (n = 1260, 62.5%) were subjected to medical therapy or successful CTO-PCI. We performed a propensity score matching (PSM) to balance the baseline characteristics. A comparison of the major adverse cardiac events (MACE) was done to evaluate long-term outcomes.

**Results:**

The median follow-up duration was 2.6 years. Through multivariate analysis, the incidence of MACE was significantly higher among diabetic patients compared to the non-diabetic patients (adjusted hazard ratio [HR] 1.32, 95% confidence interval [CI] 1.09–1.61, p = 0.005). Among the diabetic group, the rate of MACE (adjusted HR 0.61, 95% CI 0.42–0.87, p = 0.006) was significantly lower in the successful CTO-PCI group than in the MT group. Besides, in the non-diabetic group, the prevalence of MACE (adjusted HR 0.85, 95% CI 0.64–1.15, p = 0.294) and cardiac death (adjusted HR 0.94, 95% CI 0.51–1.70, p = 0.825) were comparable between the two groups. Similar results as with the early detection were obtained in propensity-matched diabetic and non-diabetic patients. Notably, there was a significant interaction between diabetic or non-diabetic with the therapeutic strategy on MACE (p for interaction = 0.036).

**Conclusions:**

For treatment of CTO, successful CTO-PCI highly reduces the risk of MACE in diabetic patients when compared with medical therapy. However, this does not apply to non-diabetic patients.

## Background

Epidemiology reports have projected that the global number of patients with diabetes mellitus (DM) will increase to 360 million by 2030 [1]. Patients with DM experience a greater atherosclerotic burden, higher rate of complex coronary arterial disease (CAD), higher risks of developing postoperative complications, and adverse outcomes after revascularization compared with non-diabetic patients [2, 3].

Coronary chronic total occlusions (CTOs) occur in 18–30% of all diagnostic coronary angiography and pose serious obstacles in the coronary intervention [4, 5]. Successful percutaneous coronary intervention (PCI) of CTOs has been reported to reduce angina, and improve both long-term survival and ventricular function compared to unsuccessful revascularization [6–9]. However, CTO-PCI can be conducted in 10–20% patients because its procedures are associated with complex lesions, a higher likelihood of procedure failure rates, and risk of major complications compared to the intervention of non-CTO lesions [4, 10, 11]. Therefore, most CTO patients receive medication rather than PCI [12, 13].

Furthermore, previous studies have reported that approximately 34 to 40% of patients with CTOs have DM [4, 14]. However, there is a paucity of data on whether the clinical outcome of revascularization and medical therapy (MT) differ for diabetic and non-diabetic CTO patients. Moreover, the studies mainly report on outcomes of successful and failed PCI in CTO patients but rarely considered the patients who received MT without CTO-PCI attempt [15, 16]. Therefore, we sought to compare the clinical outcomes of MT with successful CTO-PCI in CTO patients with and without type 2 diabetes mellitus.

## Methods

### Study population

**Fig. 1:**
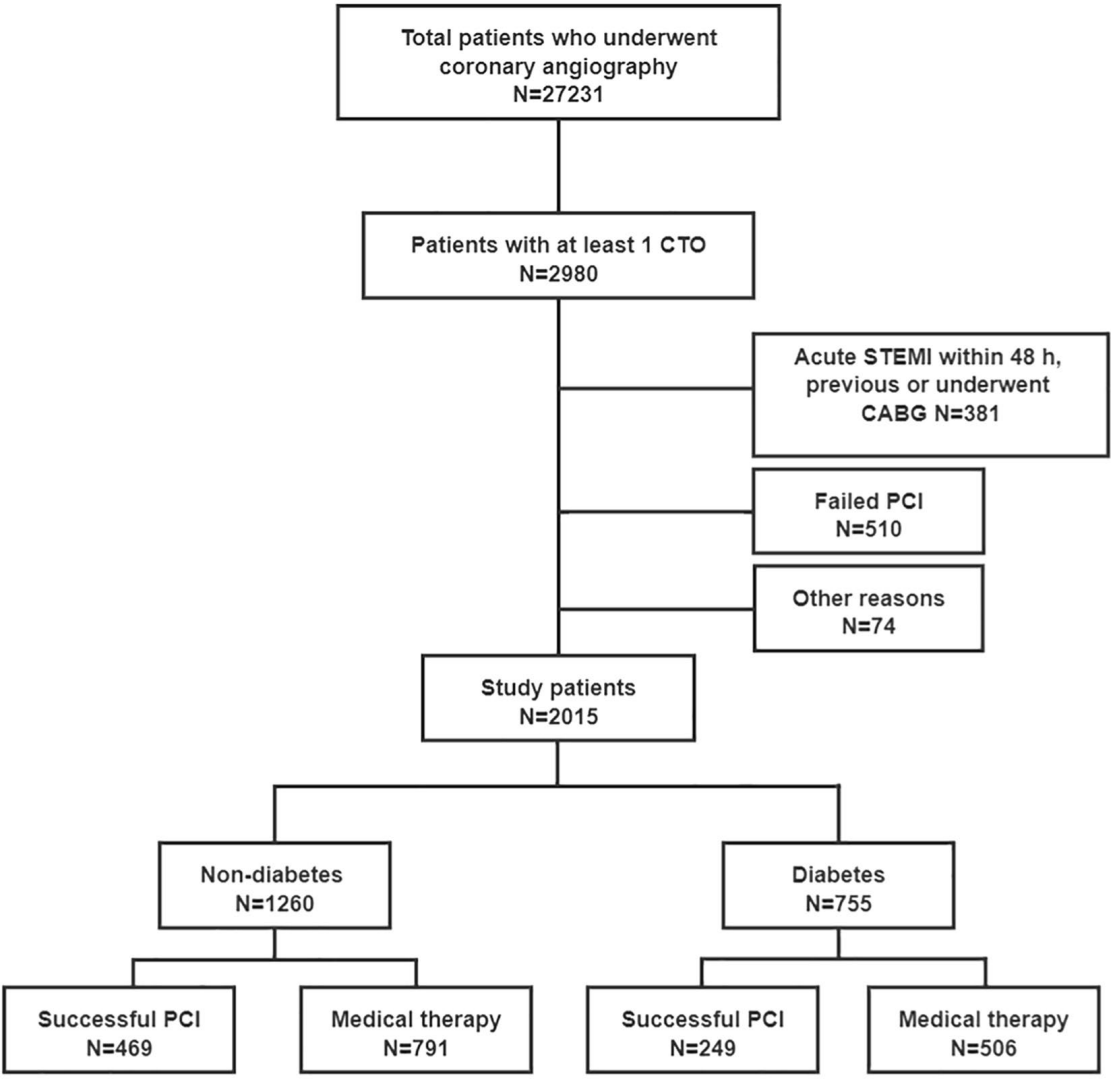
Study flow chart. *CABG* coronary artery bypass grafting, *CTO* chronic total occlusion, *PCI* percutaneous coronary intervention, *STEMI* ST-segment elevation myocardial infarction

Coronary angiography was performed in 27,231 consecutively patients at our center from January 2007 to December 2018. Notably, we included 2980 (10.9%) patients who had at least 1 CTO case. The exclusion criterion was as follows: Patients with ST-segment elevation myocardial infarction (STEMI), have a history of coronary artery bypass grafting (CABG), have type 1 diabetes, underwent failed CTO-PCI or CABG, have a history of cardiogenic shock or had a malignant tumor. After the exclusion, 755 (37.5%) patients with type 2 DM and 1260 (62.5%) patients without DM were enrolled for the final analysis. Each study group was categorized into 2 groups (successful CTO-PCI or optimal MT) following the initial treatment strategy on an intention-to-treat (ITT) basis (Fig. 1). Patients referred for PCI showed symptomatic angina, and/or myocardial viability in the territory of CTO or inducible ischemia [11, 13]. These were assessed by cardiac magnetic resonance imaging, dimensional echocardiography, or myocardial perfusion scan [11, 13, 17]. Demographic, angiographic, and procedural data were collected by reviewing hospital records and dedicated database. Follow up on the patients was conducted by reviewing hospital readmission records, telephone interviews, or outpatient visits. The patients’ personal information was kept confidential. This study was approved by our institutional review board.

### Medical treatment and PCI procedure

For medical therapy, antiplatelet medication, statins, renin-angiotensin system blockade, β-blockers, and nitrate were used. Coronary interventions were performed following current standard guidelines. All patients were pre-treated with aspirin and clopidogrel before catheterization. Thereafter, heparin (70–100 IU/kg) was administered before PCI, however, the use of glycoprotein IIb/IIIa inhibitors was at the physician’s discretion. Dual-antiplatelet medication was administered to the patients after PCI for at least 12 months.

### Study definitions and follow-up

We defined Diabetes Mellitus as a fasting glucose level ≥ 7.0 mmol/L or a glucose level ≥ 11.1 mmol/L at 2 h after a meal on more than two occasions, or the current use of oral hypoglycemic agents or insulin [18]. Besides, a “CTO lesion” was defined as a complete occlusion with anterograde Thrombolysis In Myocardial Infarction (TIMI) flow grade of 0 for more than 3 months [19]. The duration was determined based on clinical history or previous angiography. Further, we assessed the major adverse cardiac event (MACE) as a “primary endpoint” consisting of cardiac mortality, myocardial infarction (MI), or target vessel revascularization (TVR). The “secondary endpoint” was cardiac mortality. We defined the angiographic success of CTO-PCI as the restoration of TIMI grade 3 flow with residual stenosis of less than 20% after implanting a drug-eluting stent to the CTO vessel. Cardiac mortality, MI, and TVR were defined as Standardized Definitions [20].

### Statistical analysis

Data for continuous variables were presented as mean ± standard deviation whereas, data for categorical variables were presented as percentages. The Student’s t-test was used to compare differences between groups for continuous variables, whereas Chi-square or the Fisher exact test was used for discrete variables. Kruskal–Wallis test was used to compare non-parametric data. Survival-free of adverse events was determined through Kaplan–Meier analysis and compared using the log-rank test. A multivariable Cox regression model was generated, whereby covariates with either p values < 0.1 on the univariate analysis or potential clinically relevant factors including age, sex, smoking, hypertension, dyslipidemia, history of MI, heart failure, chronic kidney disease (CKD), left ventricular ejection fraction (LVEF), left anterior descending artery (LAD) involvement, multivessel disease, Japanese-chronic total occlusion (J-CTO) score, and SYNTAX score were considered as candidate variables. Additionally, we constructed a propensity score matching (PSM) to balance the baseline characteristics using the multivariable logistic regression model. The variables used in the PSM are shown in Table 2. The nearest neighbor matching algorithm was used for PSM via a 1: 2 matching protocol. All tests were performed at a 0.05 level. The SPSS Version 24.0 (SPSS Inc., Chicago, Illinois, USA) and Stata Version 15.1 (StataCorp LLC, TX, USA) was used for all statistical computations.

## Results

### Characteristics of the study patients

The prevalence of CTO was 10.9% in the total population. Notably, out of the 2015 patients with CTOs, 755 (37.5%) patients had type 2 diabetes whereas, 315 (41.7%) patients had insulin-dependent DM. The baseline demographic, angiographic, and procedural characteristics of the patients with and without DM are shown in Table 1. Moreover, patients in the DM group were older and exhibited a higher percentage of hypertension, dyslipidemia, prior MI, CKD and heart failure, more extensive coronary artery disease, lower LVEF, and higher prevalence of female gender than patients in the non-diabetic group. Smoking and CTO of LAD were more common in the non-diabetic group. We did not observe a significant difference in the prevalence of in-hospital death.

**Table 1:** Baseline clinical, angiographic, and procedural characteristics and in-hospital outcome of all patients with and without diabetes, and of all patients with and without diabetes stratified according to medical therapy or successful CTO-PCI

Variables	Total population		P value	Patients with diabetes		P value	Patients without diabetes		P value
	Diabetes	Non-diabetes		MT	Successful PCI		MT	Successful PCI	
	(n = 755)	(n = 1260)		(n = 506)	(n = 249)		(n = 791)	(n = 469)	
Age, years	65.1 ± 9.8	63.8 ± 10.7	0.022	65.6 ± 10.2	64.1 ± 8.9	0.081	64.3 ± 11.0	62.9 ± 10.1	0.025
Male	527 (69.8)	1021 (81.0)	< 0.001	357 (70.6)	170 (68.3)	0.521	650 (82.2)	371 (79.1)	0.179
Smoking	275 (36.4)	583 (46.3)	< 0.001	188 (37.2)	87 (34.9)	0.237	364 (46.0)	219 (46.7)	0.816
Hypertension	572 (75.8)	803 (63.7)	< 0.001	389 (76.9)	183 (73.5)	0.308	508 (64.2)	295 (62.9)	0.637
Dyslipidemia	585 (77.5)	900 (71.4)	0.015	399 (78.9)	186 (74.7)	0.191	565 (71.4)	335 (71.4)	0.740
Familial history of CAD	63 (8.3)	164 (13.0)	0.001	44 (8.7)	19 (7.6)	0.619	100 (12.6)	41 (14.4)	0.609
Previous MI	309 (40.9)	383 (30.4)	< 0.001	215 (42.5)	94 (37.8)	0.213	271 (34.3)	112 (23.9)	< 0.001
CKD	91 (12.1)	102 (8.1)	< 0.001	67 (13.2)	24 (9.6)	0.138	75 (9.5)	27 (5.8)	0.019
Heart failure	159 (21.1)	181 (14.4)	0.004	110 (21.7)	49 (19.7)	0.514	140 (17.7)	41 (8.7)	< 0.001
LVEF	52.0 ± 10.5	53.2 ± 9.2	0.010	51.4 ± 11.1	53.3 ± 9.1	0.320	52.3 ± 9.6	54.8 ± 8.1	< 0.001
Insulin-dependent DM	315 (41.7)	–	–	219 (43.3)	96 (38.6)	0.208	–	–	–
Baseline medication									
Aspirin	727 (96.3)	1216 (96.5)	0.800	484 (95.7)	243 (97.6)	0.185	759 (96.0)	457 (97.4)	0.165
Clopidogrel	704 (93.2)	1181 (93.7)	0.668	467 (92.3)	237 (95.2)	0.137	723 (91.4)	458 (97.7)	< 0.001
Statin	726 (96.2)	1205 (95.6)	0.337	487 (96.2)	239 (96.0)	0.861	756 (95.6)	449 (95.7)	0.893
β blocker	562 (74.4)	957 (76.0)	0.445	382 (75.5)	180 (72.3)	0.343	591 (74.7)	366 (78.0)	0.182
ACEI or ARB	506 (67.0)	780 (61.9)	0.021	358 (70.8)	148 (59.4)	0.002	493 (62.3)	287 (61.2)	0.689
One CTO lesion	643 (85.2)	1104 (87.6)	0.116	434 (85.8)	209 (83.9)	0.505	697 (88.1)	407 (86.8)	0.486
Two CTO lesions	106 (14.0)	144 (11.4)	0.085	68 (13.4)	38 (15.3)	0.498	87 (11.0)	57 (12.2)	0.533
LAD	237 (31.4)	452 (35.9)	0.040	137 (27.1)	100 (40.2)	< 0.001	262 (33.1)	190 (40.5)	0.008
LCX	243 (32.2)	348 (27.6)	0.029	182 (36.0)	61 (24.5)	0.002	243 (30.7)	105 (22.4)	0.001
RCA	377 (49.9)	604 (47.9)	0.385	259 (51.2)	118 (47.4)	0.327	379 (47.9)	225 (48.0)	0.983
Multivessel disease	630 (83.4)	981 (77.9)	0.002	437 (86.4)	193 (77.7)	0.002	683 (86.3)	298 (63.5)	< 0.001
Proximal or mid	514 (68.1)	909 (72.1)	0.053	336 (66.4)	187 (71.5)	0.159	554 (70.0)	355 (75.7)	0.030
CTO location									
Blunt stump	313 (41.5)	556 (44.1)	0.241	259 (51.2)	54 (21.7)	< 0.001	408 (51.6)	148 (31.6)	< 0.001
Calcification	141 (18.7)	220 (17.5)	0.491	109 (21.5)	32 (12.9)	0.004	158 (20.0)	63 (13.2)	0.002
Bending > 45°	337 (44.6)	549 (43.6)	0.641	221 (43.7)	116 (46.9)	0.449	363 (45.9)	186 (39.7)	0.031
Length ≥ 20 mm	477 (63.2)	810 (64.3)	0.617	320 (63.2)	157 (63.1)	0.960	505 (63.8)	305 (65.0)	0.670
J-CTO score	1.66 ± 1.16	1.67 ± 1.17	0.930	1.78 ± 1.23	1.41 ± 0.98	< 0.001	1.80 ± 1.24	1.47 ± 1.03	< 0.001
SYNTAX score	23.6 ± 8.7	21.1 ± 8.3	0.044	24.6 ± 9.1	21.5 ± 7.5	0.308	22.6 ± 8.8	18.9 ± 6.9	0.003
Number of stents	–	–	–	–	1.46 ± 0.76	–	–	1.92 ± 0.99	–
Total stent length, mm	–	–	–	–	42.1 ± 23.1	–	–	23.8 ± 24.2	–
Contrast volume, ml	175 ± 76	179 ± 85	0.844	150 ± 64	228 ± 72	< 0.001	151 ± 74	226 ± 83	< 0.001
Coronary dissection	–	–	–	–	11 (4.4)	–	–	15 (3.2)	–
Coronary perforation	–	–	–	–	2 (0.8)	–	–	6 (1.3)	–
In-hospital death	5 (0.7)	5 (0.4)	0.622	–	–	–	–	–	–

Values are presented as the mean ± standard deviation or n (%)

*ACEI* angiotensin-converting enzyme inhibitor, *ARB* angiotensin-receptor blocker, *CAD* coronary artery disease, *CKD* chronic kidney disease, *CTO* chronic total occlusion, *DM* diabetes mellitus, *J*-*CTO* Japanese-chronic total occlusion, *LAD* left ascending coronary artery, *LCX* left circumflex coronary artery, *LVEF* left ventricular ejection fraction, *MI* myocardial infarction, *MT* medical therapy, *PCI* percutaneous coronary intervention, *RCA* right coronary artery

In the diabetic group, 506 patients received MT while 249 patients underwent successful CTO-PCI. Notably, patients who underwent successful procedures more often had CTO of LAD and were less likely to develop the multivessel disease, left circumflex coronary artery (LCX) CTO, lesions of calcification, blunt stump, and J-CTO score compared to patients in the MT group.

In the non-diabetic group, 469 patients underwent successful CTO-PCI while 791 patients received MT. Patients who underwent successful CTO-PCI were younger and showed fewer cases of previous MI, CKD, and heart failure, but higher LVEF compared to patients in the MT group. Regarding angiographic characteristics, successful CTO-PCI group exhibited fewer cases of multivessel disease, LCX CTO, bending > 45°, calcification, blunt stump, high J-CTO score, and SYNTAX score than the MT group. However, LAD CTO and proximal or mid-CTO locations were more common among patients with successful CTO-PCI arm procedure.

Moreover, in the diabetic group, 270 patients were subjected to MT while 135 patients underwent successful CTO-PCI after PSM. Of note, baseline characteristics were not significantly different between both matched groups. Besides, in the non-diabetic group, 464 patients received MT while 232 patients underwent successful CTO-PCI. Similarly, we did not find considerable differences in the baseline clinical and lesion characteristics among the two matched groups, except for multivessel disease (Table 2).

**Table 2:** Baseline clinical, angiographic and procedural characteristics of propensity-matched patients with and without diabetes stratified according to medical therapy or successful CTO-PCI

	Propensity-matched patients with diabetes			Propensity-matched patients without diabetes		
	Medical therapy	Successful PCI	P value	Medical therapy	Successful PCI	P value
	(n = 270)	(n = 135)		(n = 464)	(n = 232)	
Age, years	65.2 ± 4.7	64.6 ± 9.1	0.648	63.7 ± 10.9	63.2 ± 9.9	0.340
Male	187 (69.3)	100 (74.1)	0.315	372 (80.5)	184 (79.7)	0.787
Smoking	102 (37.8)	55 (40.7)	0.564	65 (14.1)	26 (11.3)	0.301
Hypertension	197 (73.0)	96 (71.1)	0.694	302 (65.4)	151 (65.4)	1.000
Insulin-dependent DM	106 (39.3)	50 (37.0)	0.665	–	–	–
Dyslipidemia	213 (78.9)	102 (75.6)	0.447	330 (71.4)	162 (70.1)	0.722
Familial history o CAD	23 (8.5)	12 (8.9)	0.900	118 (25.5)	56 (24.2)	0.710
Previous MI	115 (42.6)	59 (43.7)	0.831	79 (17.1)	40 (17.3)	0.943
CKD	33 (12.2)	15 (11.1)	0.744	37 (8.0)	15 (6.5)	0.475
Heart failure	60 (22.2)	30 (22.2)	1.000	57 (12.3)	21 (9.1)	0.202
LVEF, %	52.1 ± 10.9	52.4 ± 9.8	0.764	54.0 ± 8.3	54.9 ± 7.6	0.876
Baseline medication						
Aspirin	265 (98.1)	132 (97.8)	0.801	451 (97.6)	224 (97.0)	0.612
Clopidogrel	256 (94.8)	128 (94.8)	1.000	442 (95.7)	227 (98.3)	0.078
Statin	256 (94.8)	131 (97.0)	0.306	441 (95.5)	221 (95.7)	0.897
β blocker	196 (72.6)	103 (76.3)	0.424	369 (79.9)	188 (81.4)	0.636
ACEI or ARB	172 (63.7)	85 (63.0)	0.884	289 (62.6)	142 (61.5)	0.782
One CTO lesion	235 (87.0)	115 (85.2)	0.608	392 (84.8)	192 (83.1)	0.555
Two CTO lesions	33 (12.2)	19 (14.1)	0.599	63 (13.6)	35 (15.2)	0.589
LAD	85 (31.5)	51 (37.8)	0.206	175 (37.9)	89 (38.5)	0.868
LCX	73 (27.0)	38 (28.1)	0.813	120 (26.0)	62 (26.8)	0.807
RCA	143 (53.0)	62 (45.9)	0.182	232 (50.2)	113 (48.9)	0.747
Multivessel disease	221 (81.9)	105 (77.8)	0.329	360 (77.9)	154 (66.7)	0.001
Proximal or mid	170 (63.0)	94 (69.6)	0.184	341 (73.8)	170 (73.6)	0.159
CTO location						
Blunt stump	85 (31.5)	33 (24.4)	0.142	183 (39.6)	74 (32.0)	0.052
Calcification	32 (11.9)	17 (12.6)	0.829	71 (15.4)	38 (16.5)	0.712
Bending > 45°	117 (43.3)	58 (43.0)	0.943	206 (44.6)	86 (37.2)	0.064
Length ≥ 20 mm	160 (59.3)	85 (63.0)	0.472	306 (66.2)	141 (61.0)	0.178
J-CTO score	1.43 ± 1.16	1.41 ± 0.98	0.216	1.63 ± 1.11	1.44 ± 1.05	0.139
SYNTAX score	23.7 ± 8.3	22.4 ± 7.9	0.658	24.2 ± 8.5	22.7 ± 7.8	0.504

Values are presented as the mean ± standard deviation or n (%)

*ACEI* angiotensin-converting enzyme inhibitor, *ARB* angiotensin-receptor blocker, *CAD* coronary artery disease, *CKD* chronic kidney disease, *CTO* chronic total occlusion, *J*-*CTO* Japanese-chronic total occlusion, *LAD* left ascending coronary artery, *LCX* left circumflex coronary artery, *LVEF* left ventricular ejection fraction, *MI* myocardial infarction, *PCI* percutaneous coronary intervention, *RCA* right coronary artery

### Follow-up outcomes

**Fig. 2:**
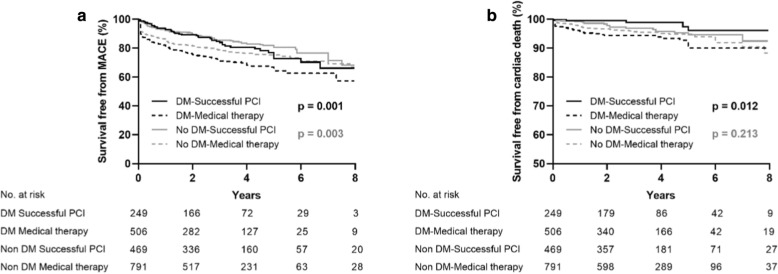
Kaplan–Meier curves for MACE (**a**) and cardiac death (**b**) during follow-up for successful CTO-PCI versus medical therapy in total patients with and without diabetes. *CTO* chronic total occlusion, *DM* diabetes mellitus, *MACE* major adverse cardiovascular events, *PCI* percutaneous coronary intervention

The median follow-up time was 2.6 (interquartile range (IQR), 1.2-4.7) years. Through multivariate analysis, we found that the MACE rate was significantly higher in the diabetic patients compared to the non-diabetic patients (diabetes vs. non-diabetes: 25.4% vs. 20.4%, adjusted hazard ratio [HR] 1.32, 95% confidence interval [CI] 1.09–1.61, p = 0.005), however, the occurrence of cardiac death (diabetes vs. non-diabetes: 5.0% vs. 4.7%, adjusted HR 1.13, 95% CI 0.73–1.75, p = 0.597) was not significantly different between the diabetic and non-diabetic groups. In the diabetic group, the incidence of MACE (successful CTO-PCI vs. MT: 18.5% vs. 28.9%, adjusted HR 0.61, 95% CI 0.42–0.87, p = 0.006) and cardiac mortality (successful CTO-PCI vs. MT: 1.4% vs. 3.7%, adjusted HR 0.29, 95% CI 0.10–0.80, p = 0.017) were significantly lower in successful CTO-PCI group compared to the MT group. In the non-diabetic group, the prevalence of MACE (successful CTO-PCI vs. MT: 16.2% vs. 22.8%, adjusted HR 0.85, 95% CI 0.64–1.15, p = 0.294) and cardiac mortality (successful CTO-PCI vs. MT: 3.8% vs. 5.2%, adjusted HR 0.94, 95% CI 0.51–1.70, p = 0.825) were not significantly different between the 2 groups (Table 3, Fig. 2).

**Table 3:** Clinical outcomes of all patients with and without diabetes, and of all patients with and without diabetes stratified according to medical therapy or successful CTO-PCI

Total population	Non-diabetes	Diabetes	P value
	(n = 1260)	(n = 755)	
Cardiac death	59 (4.7)	38 (5.0)	
Adjusted HR (95% CI)	1	1.13 (0.73–1.75)	0.597
MI	86 (6.8)	69 (9.1)	
Adjusted HR (95% CI)	1	1.38 (0.98–1.92)	0.058
TVR	155 (12.3)	113 (15.0)	
Adjusted HR (95% CI)	1	1.41 (1.10–1.81)	0.006
MACE	257 (20.4)	192 (25.4)	
Adjusted HR (95% CI)	1	1.32 (1.09–1.61)	0.005

Patients with diabetes	Medical therapy	Successful PCI	P value
	(n = 506)	(n = 249)	
Cardiac death	33 (6.5)	5 (2.0)	
Adjusted HR (95% CI)	1	0.29 (0.10–0.80)	0.017
MI	50 (9.9)	19 (7.6)	
Adjusted HR (95% CI)	1	0.75 (0.44–1.30)	0.305
TVR	79 (15.6)	34 (13.7)	
Adjusted HR (95% CI)	1	0.76 (0.50–1.17)	0.212
MACE	146 (28.9)	46 (18.5)	
Adjusted HR (95% CI)	1	0.61 (0.42–0.87)	0.006

Patients with diabetes	Medical therapy	Successful PCI	P value
	(n = 791)	(n = 469)	
Cardiac death	41 (5.2)	18 (3.8)	
Adjusted HR (95% CI)	1	0.94 (0.51–1.70)	0.825
MI	56 (7.1)	30 (6.4)	
Adjusted HR (95% CI)	1	1.01 (0.62–1.65)	0.959
TVR	109 (13.8)	46 (9.8)	
Adjusted HR (95% CI)	1	0.85 (0.59–1.22)	0.389
MACE	180 (22.8)	77 (16.4)	
Adjusted HR (95% CI)	1	0.85 (0.64–1.15)	0.294

Values are presented as n (%)

*CI* confidence interval(s), *HR* hazard ratio, *MACE* major adverse cardiovascular events, *MI* myocardial infarction, *PCI* percutaneous coronary intervention, *TVR* target-vessel revascularization

**Fig. 3:**
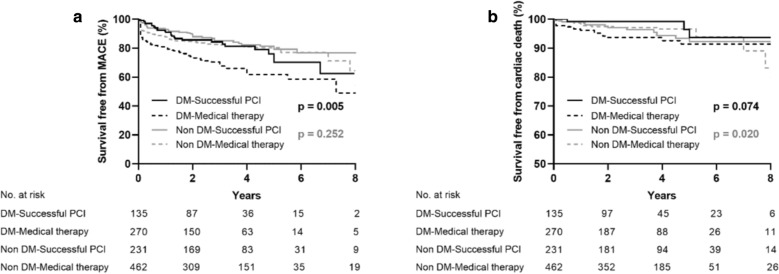
Kaplan–Meier curves for MACE (**a**) and cardiac death (**b**) during follow-up for successful CTO-PCI versus medical therapy in propensity-matched patients with and without diabetes. *CTO* chronic total occlusion, *DM* diabetes mellitus, *MACE* major adverse cardiovascular events, *PCI* percutaneous coronary intervention

In propensity-matched diabetic patients, those who received MT exhibited a higher rate of MACE (HR 0.54, 95% CI 0.35–0.84, p = 0.006) compared to patients in the successful CTO-PCI group, whereas the incidence of cardiac death (HR 0.334, 95% CI 0.10–1.17, p = 0.088) was similar between the two groups. In propensity-matched non-diabetic patients, the rate of MACE (HR 0.80, 95% CI 0.55–1.17, p = 0.257) and cardiac mortality (HR 1.05, 95% CI 0.50–2.19, p = 0.895) were not significantly different in the two groups (Table 4, Fig. 3).

**Table 4:** Clinical outcomes of propensity-matched patients with and without diabetes stratified according to medical therapy or successful CTO-PCI

Patients with diabetes	Medical therapy	Successful PCI	P value
	(n = 270)	(n = 135)	
Cardiac death	17 (6.3)	3 (2.2)	
HR (95% CI)	1	0.34 (0.10–1.17)	0.088
MI	27 (10.0)	12 (8.9)	
HR (95% CI)	1	0.88 (0.45–1.75)	0.723
TVR	48 (17.8)	19 (14.1)	
HR (95% CI)	1	0.71 (0.42–1.20)	0.202
MACE	86 (31.9)	26 (19.3)	
HR (95% CI)	1	0.54 (0.35–0.84)	0.006

Patients without diabetes	Medical therapy	Successful PCI	P value
	(n = 462)	(n = 231)	
Cardiac death	20 (4.3)	11 (4.8)	
HR (95% CI)	1	1.05 (0.50–2.19)	0.895
MI	33 (7.1)	15 (6.5)	
HR (95% CI)	1	0.91 (0.49–1.68)	0.763
TVR	55 (11.9)	23 (10.0)	
HR (95% CI)	1	0.81 (0.50–1.32)	0.400
MACE	90 (19.5)	38 (16.5)	
HR (95% CI)	1	0.80 (0.55–1.17)	0.257

Values are presented as n (%)

*CI* confidence interval(s), *HR* hazard ratio, *MACE* major adverse cardiovascular events, *MI* myocardial infarction, *PCI* percutaneous coronary intervention, *TVR* target-vessel revascularization

### An intention to treat analysis

**Fig. 4:**
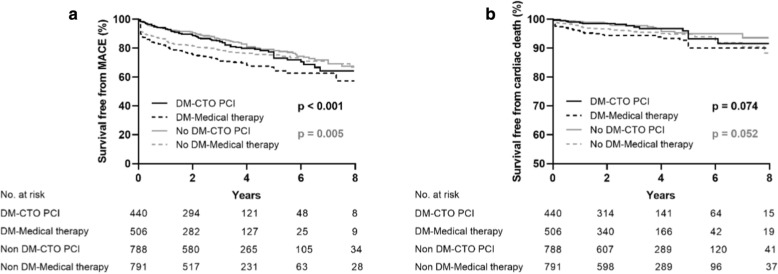
Kaplan–Meier curves for MACE (**a**) and cardiac death (**b**) during follow-up for initial CTO-PCI versus medical therapy in total patients with and without diabetes. *CTO* chronic total occlusion, *DM* diabetes mellitus, *MACE* major adverse cardiovascular events, *PCI* percutaneous coronary intervention

The baseline characteristics of medical therapy compared with initial CTO-PCI in patients with and without DM are highlighted in Additional file 1: Table S1. Notably, there were 440 diabetic patients and 788 non-diabetic patients who underwent CTO-PCI respectively following ITT. By considering the outcome of MACE, we found that initial CTO-PCI was highly beneficial to diabetic patients (adjusted HR 0.56, 95% CI 0.42–0.74, p < 0.001) compared with MT, however, it was not beneficial to the non-diabetic patients (adjusted HR 0.81, 95% CI 0.52–1.23, p = 0.297). Cases of cardiac death between diabetic (adjusted HR 0.69, 95% CI 0.37–1.28, p = 0.217) and non-diabetic patients (adjusted HR 1.05, 95% CI 0.50–2.19, p = 0.895) were not statistically significant (Additional file 1: Table S2, Fig. 4).

### Subgroup analysis

**Fig. 5:**
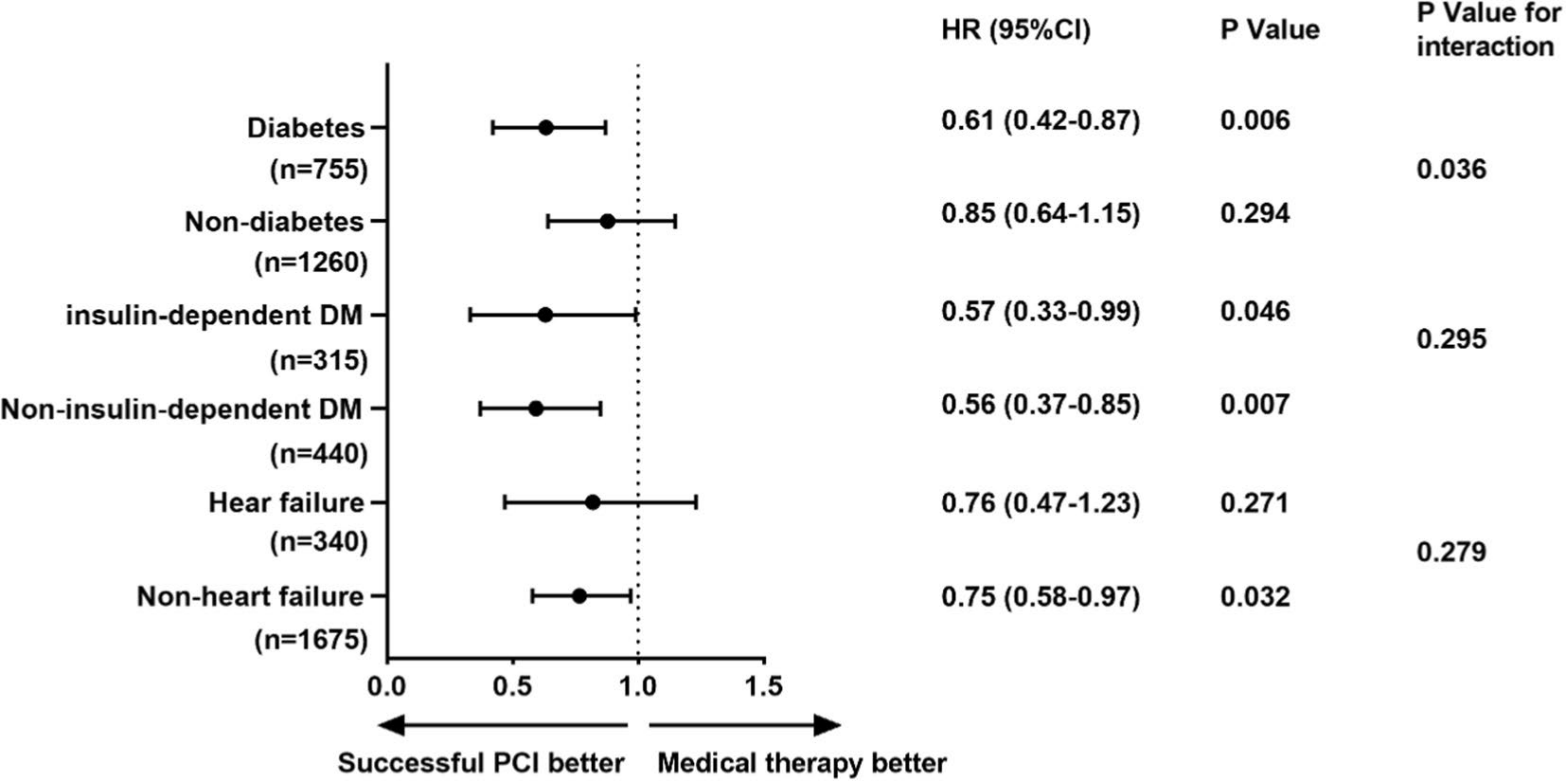
DM, insulin-dependent DM and heart failure subgroup analysis for MACE. *CI* confidence interval(s), *DM* diabetes mellitus, *HR* hazard ratio, *MACE* major adverse cardiovascular events, *PCI* percutaneous coronary intervention

Further, we noted a significant interaction between diabetic or non-diabetic conditions with therapeutic strategy following MACE (p = 0.036). The survival free from MACE benefit of successful CTO-PCI was highly significant among diabetic patients than in non-diabetic patients. Similarly, patients without cases of heart failure benefited from successful CTO-PCI for MACE, however, the effect was not observed among the patients with heart failure (p for interaction = 0.279). Additionally, the survival free from MACE benefit of successful CTO-PCI was comparable in insulin-dependent DM and non-insulin-dependent DM patients. Besides, the interaction between insulin-dependent DM or insulin-independent DM with therapeutic strategy following MACE was not significant (p = 0.295), (Fig. 5).

## Discussion

We assessed the long-term outcomes of different treatment strategies in CTO patients with and without type 2 DM in a large cohort population. Notably, we confirmed the following: (1) Diabetic patients with CTOs are highly prone to lower LVEF, multivessel disease, and complex lesions, and encounter more long-term adverse clinical outcomes compared to non-diabetic patients. (2) Successful CTO-PCI reduces MACE compared to the use of medical therapy alone in diabetic patients, this was confirmed by both the multivariable Cox regression and PSM analyses. (3) Successful CTO-PCI is not associated with reduced MACE or cardiovascular mortality in non-diabetic patients with CTOs.

In addition, DM is an independent risk factor for CAD and has been reported to be associated with longer coronary lesions, more complex anatomy, comorbidities, and more adverse cardiovascular events [21]. Similarly, we observed that diabetic patients have a significantly higher prevalence of multivessel disease and SYNTAX score compared to non-diabetic patients, this concurs with the findings by Choi and the team [22]. A previous study showed that diabetic patients receiving primary PCI had more common CTO lesions in non-infarct related compared with non-diabetic patients (21% vs. 12%) [23]. Moreover, large contemporary CTO registries have reported that 41–45% of patients undergoing CTO-PCI had DM [24, 25]. However, there are no available reports on the association of the outcomes with an optimal therapeutic strategy in DM or non-DM CTO patients. To our knowledge, this is the first report that assessed the long-term clinical outcomes of successful PCI compared with medical therapy in unselected on a large cohort of CTO patients with and without type 2 diabetes.

Besides, previous studies indicate that DM is an independent risk factor for restenosis, need for revascularization and MACEs, particularly in patients with longer coronary lesion [26, 27]. Additionally, Kandzari et al. demonstrated that diabetic patients with CTOs who underwent PCI with sirolimus-eluting stents exhibited higher rates of restenosis and TVR compared to non-diabetic patients (22% vs. 4.7%) [28], this observation is consistent with our findings. Elsewhere, Safley et al. reported similar survival rates between CTO and non-CTO diabetic patients (75% vs. 79%, p = 0.20) after 5 years of follow-up [29]. In the sub-analysis of CIBELES trial whereby 207 patients underwent successful CTO-PCI with a drug-eluting stent, the rates of cardiac events including death, MI, and TVR were comparable in diabetic and non-diabetic patients. However, this study enrolled only 75 diabetic patients with 21% insulin-dependent diabetics and the follow-up period was relatively short (12 months). This may not accurately reflect the clinical outcomes in diabetic and non-diabetic patients [3]. Contrarily, Claessen et al. reported that CTO patients with DM exhibited a higher long-term mortality rate compared to patients without DM [30]. Further, among the diabetic patients, successful CTO-PCI was associated with reduced long-term mortality and subsequent CABG [30]. Moreover, a large-scale study that included 6320 patients who underwent PCI showed that mortality is higher in diabetic patients than in non-diabetic patients [31]. In recent a meta-analysis which included 4571 patients with CTO (1915 diabetic patients and 2656 non-diabetic patients), CTO patients with DM exhibited significantly higher rates of mortality, repeated revascularization, and MACEs compared to patients without DM [32]. Likewise, our study reported that TVR and MACE rates are higher in diabetic than in non-diabetic patients, this is consistent with the finding of Rha and coworkers [33].

Diabetic patients have a great atherosclerotic burden, restenosis after PCI, and more adverse events probably because they are characterized by frequent hyperplasia after PCI, more easily activated platelets, increased levels of fibrinogen, thrombin and coagulation factor VII, proinflammatory states, systemic endothelial dysfunction, and metabolic disorders [21, 34, 35]. Of note, collateral circulation development is known to be less in diabetic patients than in non-diabetic patients when coronary arteries become occluded. Particularly, in CTO patients, well-developed coronary collateral circulation potentially supplies the downstream perfusion area and thereby alleviates myocardial ischemia, preserves viable myocardium, reduces infarct area, improves left ventricular function, and decrease cardiovascular mortality [36]. This may explain the worse outcome of CTO patients with DM [36].

Notably, previous cohort studies mainly focused on the outcomes of successful PCI as opposed to failed procedures in CTO patients with DM, thus reported different results [29, 30, 37]. However, the higher rates of procedural complications and adverse events directly associated with failed CTO procedures were rarely considered thereby contributes to the poor prognosis of CTO patients [38]. The high rate of crossovers of failed CTO-PCI and medial therapy groups limits conclusions and may underestimate the actual effect of successful CTO-PCI. Further, patients treated via medical therapy without an attempted CTO-PCI were not enrolled in the previous studies [15]. Limited reports exist on the definite evidence of improved clinical outcomes of successful CTO-PCI compared with medical treatment (CTO-PCI not attempted), and the data is urgently needed [15, 16]. Also, in the DECISION-CTO [39] and the Euro-CTO trials [40], detailed clinical outcomes of CTO patients with DM were not analyzed. Therefore, our study excluded patients who underwent failed CTO-PCI and rather investigated the clinical outcomes between successful CTO PCI and medical therapy (CTO-PCI not attempted) groups in CTO patients with and without DM. Accordingly, our study is closer to the “real world” of the clinical practice in CTO patients with and without DM compared to previous studies.

Currently, there are no widely recognized consensus or guidelines on the treatment strategy of CTO patients with DM. Also, the prognosis of successful CTO-PCI versus medical therapy in this population is unknown. Contrary to the previous findings [22], our study showed that successful CTO-PCI reduces MACE compared to MT alone in CTO patients with DM. Nevertheless, among CTO patients without DM, we did not observe a reduction in MACE or cardiovascular mortality when compared with MT alone (although the MACE rate was higher in MT group). In addition, we performed PSM to adjust for potential selection bias and the influence of confounding factors, and maintain a balance in covariates. Results concurred with earlier findings before PSM was conducted. Besides, an ITT analysis of medical therapy versus initial CTO-PCI was performed for a highly comprehensive evaluation. Of note, initial CTO-PCI was highly beneficial to diabetic patients considering MACE when compared with MT. In the randomized COURAGE trial which compared PCI with MT in patients with stable coronary heart disease, subgroup analysis did not show any beneficial clinical outcomes among nondiabetic patients [41], this concurs with our findings. Additionally, we observed a significant interaction between diabetic or non-diabetic patients and therapeutic strategy regarding MACE, this suggests that the superiority of successful CTO-PCI over MT is dependent on the glucose level.

Besides, diabetic patients who are a higher- risk group were less likely to undergo CTO-PCI compared with non-diabetic patients. However, these higher-risk patients, highly benefit from the “treatment-risk paradox”, which is a common procedure in PCI [21, 42]. Higher event rates in the high-risk subjects increase the statistical power in detecting the significant differences in adverse outcomes. These findings indicate that successful CTO-PCI has more clinical benefits in diabetic patients compared to non-diabetic patients. Besides, complete revascularization is associated with fewer MACEs and improved long-term survival in patients with multivessel disease and STEMI or angina [43, 44]. Previously, studies showed that success rates of CTO-PCI are similar in diabetic and non-diabetic patients [29, 30]. Furthermore, the two recent large CTO studies (OPEN CTO registry and PROGRESS CTO registry) in which current dedicated equipment and skills including hybrid algorithms have been applied, represent modern CTO-PCI standards. The two studies reported that high procedural success, however, similar and complication rates are low in patients with and without diabetes [24, 25]. Therefore, with the latest refinement equipment and techniques, PCI of CTO is safe, has high success rates, and poses low complication rates in patients with DM. In treating CTO patients with diabetes, CTO-PCI may be highly preferred as the treatment option.

## Study limitations

This study is observational, though, we performed PSM to adjust the potential selection bias and minimize the confounding factors. Nevertheless, diabetes is on the rise as one of the leading causes of cardiovascular mortality worldwide, therefore, our findings on the high-risk subset of patients may be particularly meaningful.

## Conclusions

Successful CTO-PCI potentially reduces the risk of MACE in diabetic patients compared to when medical therapy is used alone for treating chronic total occlusions. However, this intervention does not work for non-diabetic patients. Therefore, CTO-PCI provides a safe and effective treatment option for unselected CTO patients with diabetes. Large randomized clinical trials are thus warranted to verify these findings.

## Supplementary information

**Additional file 1: Table S1.** Baseline clinical, angiographic and procedural characteristics of patients with and without diabetes stratified according to medical therapy or initial CTO-PCI. **Table S2.** Clinical outcomes of patients with and without diabetes stratified according to medical therapy or initial CTO-PCI.

## Data Availability

The datasets generated and analyzed for this current study are available from the corresponding author upon reasonable request.

## Abbreviations

CABG: Coronary artery bypass grafting
CAD: Coronary artery disease
CTO: Chronic total occlusion
DM: Diabetes mellitus
J-CTO: Japanese-chronic total occlusion
LAD: Left anterior descending artery
LVEF: Left ventricular ejection fraction
MACE: Major adverse cardiovascular events
MI: Myocardial infarction
MT: Medical therapy
PCI: Percutaneous coronary intervention
PSM: Propensity score matching
TVR: Target-vessel revascularization
