# Correction to: Long‑term outcomes of medical therapy versus successful recanalisation for coronary chronic total occlusions in patients with and without type 2 diabetes mellitus

**DOI:** 10.1186/s12933-021-01218-5

**Published:** 2021-01-30

**Authors:** Lei Guo, Junjie Wang, Huaiyu Ding, Shaoke Meng, Xiaoyan Zhang, Haichen Lv, Lei Zhong, Jian Wu, Jiaying Xu, Xuchen Zhou, Rongchong Huang

**Affiliations:** 1 Department of Cardiology, The First Affiliated Hospital of Dalian Medical University, Dalian, People’s Republic of China; 2 Department of Radiology, Fuyang Hospital of Anhui Medical University, Fuyang, People’s Republic of China; 3 Department of Cardiology, Capital Medical University Affiliated Beijing Friendship Hospital, Beijing, People’s Republic of China

## Correction to: Cardiovasc Diabetol (2020) 19:100 https://doi.org/10.1186/s12933-020–01087-4

Following publication of the original article [1], the authors would like to include the article note, “Lei Guo and Rongchong Huang contributed equally to this work”.

Also, the affiliation for the co-author should be “Rongchong Huang^1,3^” instead of “Rongchong Huang^3^”.

The original article [1] has been updated.

## References

[1] Guo L, Wang J, Ding H, Meng S, Zhang X, Lv H, Zhong L, Wu J, Xu J, Zhou X, Huang R (2020). Long-term outcomes of medical therapy versus successful recanalisation for coronary chronic total occlusions in patients with and without type 2 diabetes mellitus. CardiovascDiabetol.

