# Supplementary material for: Long-term outcomes of medical therapy versus successful recanalisation for coronary chronic total occlusions in patients with and without type 2 diabetes mellitus
Source: Cardiovasc Diabetol. 2020 Jul 4;19:100. doi: 10.1186/s12933-020-01087-4 (PMC7335447; doi:10.1186/s12933-020-01087-4)
Supplement: Supplementary file 1 — Additional file 1: Table S1. Baseline clinical, angiographic and procedural characteristics of patients with and without diabetes stratified according to medical therapy or initial CTO-PCI. Table S2. Clinical outcomes of patients with and without diabetes stratified according to medical therapy or initial CTO-PCI. [file 12933_2020_1087_MOESM1_ESM.docx]

**Table S1** **Baseline clinical, angiographic and procedural characteristics of patients with and without diabetes stratified according to medical therapy or initial CTO-PCI.**

|  | Patients with diabetes | | | Patients without diabetes | | |
| --- | --- | --- | --- | --- | --- | --- |
|  | Medical therapy | CTO-PCI | P Value | Medical therapy | CTO-PCI | P Value |
|  | (n = 506) | (n = 440) |  | (n = 791) | (n = 788) |  |
| Age, years | 65.6 ± 10.2 | 64.3 ± 8.8 | 0.019 | 64.3 ± 11.0 | 63.0 ± 10.0 | 0.006 |
| Male | 357 (70.6) | 317 (72.0) | 0.613 | 650 (82.2) | 641 (81.3) | 0.670 |
| Smoking | 188 (37.2) | 162 (36.8) | 0.915 | 364 (46.0) | 372 (47.2) | 0.635 |
| Hypertension | 389 (76.9) | 315 (71.6) | 0.063 | 508 (64.2) | 491 (62.3) | 0.430 |
| Dyslipidemia | 399 (78.9) | 344 (78.2) | 0.786 | 565 (71.4) | 576 (73.1) | 0.346 |
| Familial history of  CAD | 44 (8.8) | 54 (12.3) | 0.072 | 100 (12.6) | 112 (14.2) | 0.360 |
| Previous MI | 215 (42.5) | 166 (37.7) | 0.136 | 271 (34.3) | 242 (30.7) | 0.132 |
| CKD | 67 (13.2) | 42 (9.5) | 0.066 | 75 (9.5) | 58 (7.4) | 0.137 |
| Heart failure | 110 (21.7) | 87 (19.8) | 0.448 | 140 (17.7) | 83 (10.5) | <0.001 |
| LVEF, % | 51.4 ± 11.1 | 52.9 ± 9.0 | 0.448 | 52.3 ± 9.6 | 54.2 ± 8.2 | <0.001 |
| Baseline medication |  |  |  |  |  |  |
| Aspirin | 484 (95.7) | 424 (96.4) | 0.578 | 759 (96.0) | 765 (97.1) | 0.222 |
| Clopidogrel | 467 (92.3) | 402 (91.4) | 0.602 | 723 (91.4) | 753 (95.6) | 0.001 |
| Statin | 487 (96.2) | 415 (94.3) | 0.160 | 756 (95.6) | 748 (94.9) | 0.543 |
| β blocker | 382 (75.5) | 301 (68.4) | 0.015 | 591 (74.7) | 599 (76.0) | 0.549 |
| ACEI or ARB | 358 (70.8) | 263 (59.8) | <0.001 | 493 (62.3) | 469 (59.5) | 0.253 |
| One CTO lesion | 434 (85.8) | 364 (82.7) | 0.199 | 697 (88.1) | 665 (84.8) | 0.032 |
| Two CTO lesions | 68 (13.4) | 72 (16.4) | 0.206 | 87 (11.0) | 113 (14.3) | 0.046 |
| LAD | 137 (27.1) | 158 (35.9) | 0.003 | 262 (33.1) | 309 (39.2) | 0.012 |
| LCX | 182 (36.0) | 117 (26.6) | 0.002 | 243 (30.7) | 194 (24.6) | 0.007 |
| RCA | 259 (51.2) | 222 (50.5) | 0.822 | 379 (47.9) | 378 (48.0) | 0.982 |
| Multivessel disease | 437 (86.4) | 348 (79.1) | 0.002 | 683 (86.3) | 566 (71.8) | <0.001 |
| Proximal or mid  CTO Location | 336 (66.4) | 314 (71.4) | 0.101 | 554 (70.0) | 587 (74.5) | 0.048 |
| Blunt stump | 259 (51.2) | 193 (43.9) | 0.025 | 408 (51.6) | 363 (46.1) | 0.028 |
| Calcification | 109 (21.5) | 76 (17.3) | 0.099 | 158 (20.0) | 113 (14.3) | 0.003 |
| Bending >45° | 221 (43.7) | 232 (52.7) | 0.005 | 363 (45.9) | 378 (48.0) | 0.408 |
| length ≥20mm | 320 (63.2) | 291 (66.1) | 0.353 | 505 (63.8) | 526 (66.8) | 0.225 |
| J-CTO score | 1.78 ± 1.23 | 1.77 ± 1.15 | 0.660 | 1.80 ± 1.24 | 1.73 ± 1.14 | 0.171 |
| SYNTAX score | 24.6 ± 9.1 | 23.5 ± 8.5 | 0.787 | 22.6 ± 8.8 | 21.1 ± 8.6 | 0.217 |

Values are presented as the mean ± standard deviation or n (%).

*ACEI* angiotensin-converting enzyme inhibitor, *ARB* angiotensin-receptor blocker, *CAD* coronary artery disease, *CKD* chronic kidney disease, *CTO* chronic total occlusion, *J-CTO* Japanese-chronic total occlusion, *LAD* left ascending coronary artery, *LCX* left circumflex coronary artery, *LVEF* left ventricular ejection fraction, *MI* myocardial infarction, *PCI* percutaneous coronary intervention, *RCA* right coronary artery

**Table S2 Clinical outcomes of patients with and without diabetes stratified according to medical therapy or initial CTO-PCI.**

| Patients with diabetes | Medical therapy | CTO-PCI | P Value |
| --- | --- | --- | --- |
|  | (n = 506) | (n = 440) |  |
| Cardiac death | 33 (6.5) | 17 (3.9) |  |
| HR (95% CI) | 1 | 0.69 (0.37-1.28) | 0.217 |
| MI | 50 (9.9) | 32 (7.3) |  |
| HR (95% CI) | 1 | 0.80 (0.50-1.26) | 0.346 |
| TVR | 79 (15.6) | 51 (11.6) |  |
| HR (95% CI) | 1 | 0.62 (0.43-0.90) | 0.013 |
| MACE | 146 (28.9) | 82 (18.6) |  |
| HR (95% CI) | 1 | 0.56 (0.42-0.74) | <0.001 |
|  |  |  |  |
| Patients without diabetes | Medical therapy | CTO-PCI | P Value |
|  | (n = 791) | (n = 788) |  |
| Cardiac death | 41 (5.2) | 26 (3.3) |  |
| HR (95% CI) | 1 | 1.05 (0.50-2.19) | 0.895 |
| MI | 56 (7.1) | 48 (6.1) |  |
| HR (95% CI) | 1 | 0.91 (0.49-1.68) | 0.763 |
| TVR | 109 (13.8) | 65 (8.2) |  |
| HR (95% CI) | 1 | 0.81 (0.50-1.32) | 0.400 |
| MACE | 180 (22.8) | 136 (17.2) |  |
| HR (95% CI) | 1 | 0.81 (0.52-1.23) | 0.297 |

Values are presented as n (%).

*CI* confidence interval(s), *HR* hazard ratio, *MACE* major adverse cardiovascular events, *MI* myocardial infarction, *PCI* percutaneous coronary intervention, *TVR* target-vessel revascularization
